# Mineralized Cartilage and Bone-Like Tissues in Chondrichthyans Offer Potential Insights Into the Evolution and Development of Mineralized Tissues in the Vertebrate Endoskeleton

**DOI:** 10.3389/fgene.2021.762042

**Published:** 2021-12-22

**Authors:** Oghenevwogaga J. Atake, B. Frank Eames

**Affiliations:** Department of Anatomy, Physiology, and Pharmacology, University of Saskatchewan, Saskatoon, SK, Canada

**Keywords:** chondrichthyan endoskeleton, sharks, skates, vertebrate mineralization patterns, skeletal evolution and development (EvoDevo), molecular fingerprints, homology

## Abstract

The impregnation of biominerals into the extracellular matrix of living organisms, a process termed biomineralization, gives rise to diverse mineralized (or calcified) tissues in vertebrates. Preservation of mineralized tissues in the fossil record has provided insights into the evolutionary history of vertebrates and their skeletons. However, current understanding of the vertebrate skeleton and of the processes underlying its formation is biased towards biomedical models such as the tetrapods mouse and chick. Chondrichthyans (sharks, skates, rays, and chimaeras) and osteichthyans are the only vertebrate groups with extant (living) representatives that have a mineralized skeleton, but the basal phylogenetic position of chondrichthyans could potentially offer unique insights into skeletal evolution. For example, bone is a vertebrate novelty, but the internal supporting skeleton (endoskeleton) of extant chondrichthyans is commonly described as lacking bone. The molecular and developmental basis for this assertion is yet to be tested. Subperichondral tissues in the endoskeleton of some chondrichthyans display mineralization patterns and histological and molecular features of bone, thereby challenging the notion that extant chondrichthyans lack endoskeletal bone. Additionally, the chondrichthyan endoskeleton demonstrates some unique features and others that are potentially homologous with other vertebrates, including a polygonal mineralization pattern, a trabecular mineralization pattern, and an unconstricted perichordal sheath. Because of the basal phylogenetic position of chondrichthyans among all other extant vertebrates with a mineralized skeleton, developmental and molecular studies of chondrichthyans are critical to flesh out the evolution of vertebrate skeletal tissues, but only a handful of such studies have been carried out to date. This review discusses morphological and molecular features of chondrichthyan endoskeletal tissues and cell types, ultimately emphasizing how comparative embryology and transcriptomics can reveal homology of mineralized skeletal tissues (and their cell types) between chondrichthyans and other vertebrates.

## The Basal Phylogenetic Position of Chondrichthyans can Provide Unique Insights Into Endoskeletal Evolution Among Vertebrates

Many organisms utilize biominerals to harden the deep (endo-) or more superficial (exo-) supporting skeleton through a process termed biomineralization. Specialized cell types (generally referred to as scleroblasts) drive biomineralization by synthesizing and secreting both the biominerals and the organic extracellular matrices into which they are incorporated (Moss, 1964; Francillon-Vieillot et al., 1990; Checa, 2018). In vertebrates, biomineralization occurs by deposition of biological apatite into collagen-/amelogenin-rich matrices, and this process gives rise to the main types of mineralized (or calcified) tissues: bone, mineralized cartilage, dentine, enamel, and enameloid (Hall, 1975; Kemp, 1989; Donoghue et al., 2006). Given that these mineralized tissue types were already distinct in ancestral vertebrates, later-diverged vertebrate groups mostly modified ancestral mineral and organic components in order to mineralize their skeletal tissues (Enlow and Brown, 1958; Francillon-Vieillot et al., 1990).

The representation of mineralized tissues in the fossil record has fleshed out the evolutionary history of vertebrates (Janvier, 1996). Chondrichthyans (sharks, skates, rays, and chimaeras) and osteichthyans (bony fishes and tetrapods) are the only vertebrate groups with extant (living) representatives that have a mineralized skeleton. Paleontological and molecular analyses have led to the recognition of chondrichthyans as phylogenetically basal to all living jawed vertebrates (Janvier, 1996; Venkatesh et al., 2001; Kikugawa et al., 2004). Thus, the basal phylogenetic position of chondrichthyans makes them excellent model organisms for revealing the evolution of mineralized endoskeletal tissues among vertebrate groups. Extant chondrichthyans are subdivided into two groups: elasmobranchs (sharks, skates, and rays) and holocephalans (chimaeras), which last shared a common ancestor at least 385 million years ago (Janvier and Pradel, 2015; Frey et al., 2019; Cohen et al., 2021). Despite their predominantly cartilaginous endoskeleton, chondrichthyans exhibit a great diversity of derived and ancestral mineralized tissues. For example, tesserae, which are discrete blocks of mineralized tissue lining endoskeletal elements, are a derived and unique skeletal feature of chondrichthyans (Kemp and Westrin, 1979; Dean and Summers, 2006). The centrum (i.e., vertebral body) of chimaeras exhibits an unconstricted perichordal sheath, considered an ancestral vertebrate feature (Miles, 1970; Schmitz, 1998). On the other hand, structural and developmental features of chondrichthyan teeth are considered homologous to those of other vertebrates and likely reflect the ancestral state of jawed vertebrates (Gillis and Donoghue, 2007; Rasch et al., 2016; Rücklin et al., 2021). In this review, we consider how recent analyses of chondrichthyan tesserae and centra shed light upon the evolution of mineralized tissues in the vertebrate endoskeleton, including examining whether chondrichthyans make bone, but first we briefly summarize some basic concepts in skeletal biology (mostly from studies of tetrapods).

## Mineralization Patterns and Developmental Processes of Bone and Cartilage

A common approach to characterize bone and generally the morphological diversity of vertebrate mineralized tissues is based on their spatial patterns (herein referred to as mineralization patterns). Mineralization patterns of the vertebrate skeleton are often described at the gross anatomical level (i.e., patterns of skeletal elements across the whole skeleton), at the macro- or micro-structural level (i.e., patterns of discrete skeletal tissues), and at the nanostructural level (e.g., patterns of collagen fibrils) (Francillon-Vieillot et al., 1990; Rho et al., 1998). Mineralization patterns at the gross anatomical and nanostructural levels have been reviewed elsewhere (Kemp, 1984; Maisey, 1988; Katz et al., 1989; Huysseune and Sire, 1998; Wiesmann et al., 2005), so we will focus mainly on mineralization patterns of skeletal tissues, particularly that of bone and mineralized cartilage.

**FIGURE 1:**
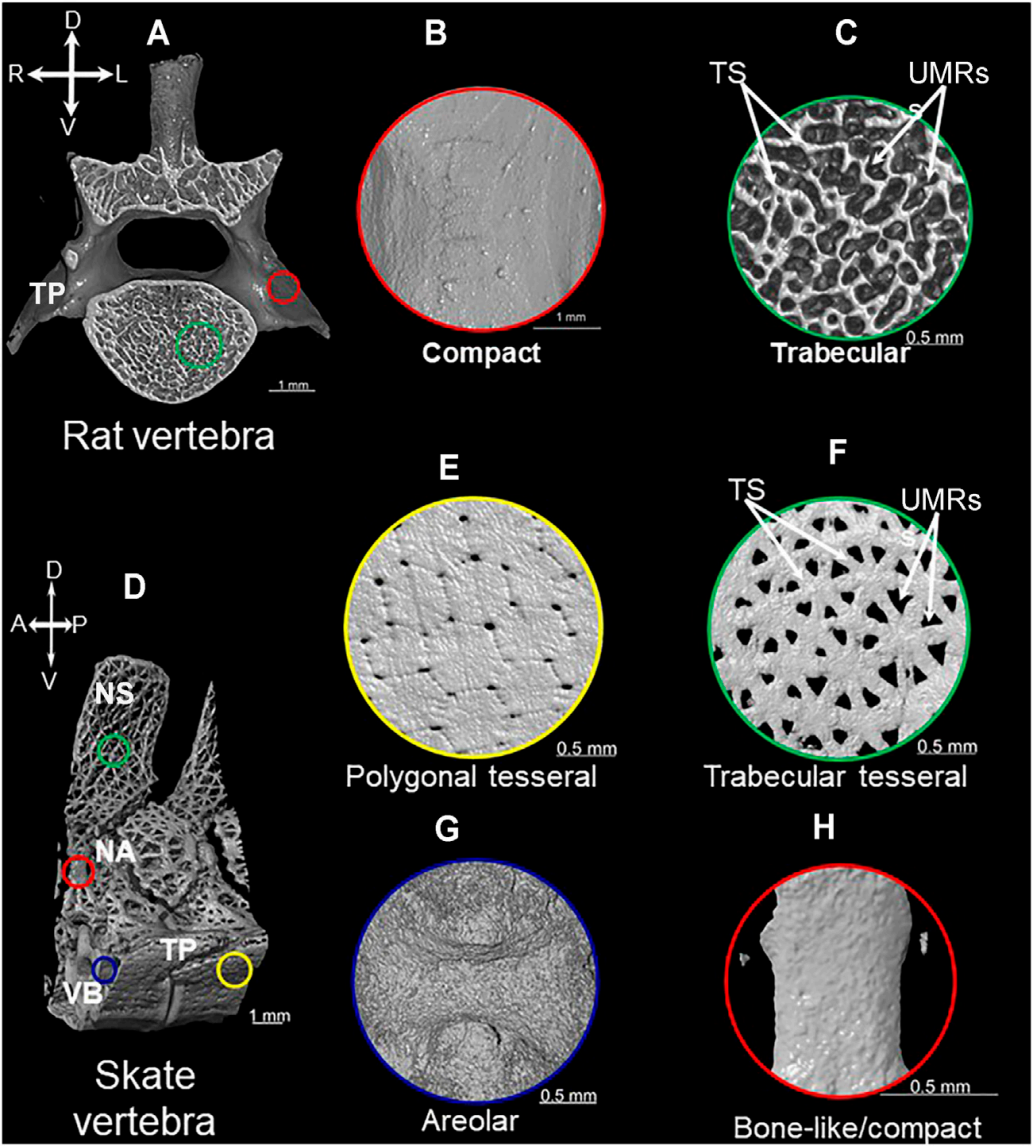
Mineralization patterns of vertebrate mineralized tissues. 3D rendered images of micro-CT scan of vertebrae from rat **(A)** and little skate (*Leucoraja erinacea*) **(D)** showing compact **(B)**, trabecular **(C)**, polygonal tesseral **(E)**, trabecular tesseral **(F)**, areolar **(G)**, and bone-like/compact **(H)** mineralization patterns. Abbreviations: TS = trabecular struts; UMRs = unmineralized regions; NS = neural spine; NA = neural arch; TP = transverse process; VB = vertebral body; A = anterior; P = posterior; R = right; L = left.

Bone is a pervasive endoskeletal tissue that exhibits two basic mineralization patterns: compact and trabecular (Francillon-Vieillot et al., 1990; Rho et al., 1998). The compact mineralization pattern is continuous and smooth, whereas the trabecular pattern has many branching, rod-like struts with unmineralized regions between them (Figures 1A–C). Compact and trabecular mineralization patterns are commonly used to characterize the microstructure of bone, but they also can apply to other vertebrate mineralized tissues, such as dentine or mineralized cartilage (Ørvig, 1951; Sire and Huysseune, 2003).

Osteoblasts are the scleroblast type that form vertebrate bone, and the process of bone formation can be either direct from isolated mesenchyme (intramembranous ossification) or indirect using a cartilage template (endochondral and/or perichondral ossification) (Olsen et al., 2000; Galea et al., 2021). In a polarized fashion, osteoblasts synthesize osteoid, the organic component of bone extracellular matrix (ECM), which contains abundant type 1 collagen (Col1) (Rossert and de Crombrugghe, 2002). Osteoblasts also secrete vesicles that initiate bone ECM mineralization (Anderson et al., 2005; Golub, 2009). Osteoblasts are usually trapped in bone ECM where they mature into osteocytes (Franz-Odendaal et al., 2006), but some bony fishes (e.g., teleosts) have acellular (anosteocytic) bone, where osteoblasts are located at the bone ECM surface. In cellular bone, osteocytes extend cytoplasmic extensions through the bone ECM in a network of nano-channels (called canaliculi) that act as mechanosensors and communication channels between neighbouring osteocytes (Aarden et al., 1994; Kamioka et al., 2001; Kerschnitzki et al., 2011).

Compared to bone, mineralized cartilage is less abundant in most vertebrate endoskeletons, found in such places as the growth plate of bones forming by endochondral ossification, articular surfaces between bones, medial portions of reptilian and mammalian ribs, and mammalian thyroid cartilages (Lohmander and Hjerpe, 1975; Ballock and O’Keefe, 2003; Claassen et al., 2017). In growth plates and thyroid cartilages, mineralized cartilage can exhibit a trabecular mineralization pattern containing wave-like running lines (termed Liesegang lines) resulting from rhythmic deposition of biological apatite in the cartilage ECM (Gerstenfeld and Shapiro, 1996; Kimpel et al., 1999; Sawae et al., 2003; Claassen et al., 2014; Jaroszewicz et al., 2016; Claassen et al., 2017; Estefa et al., 2021). Specifically, the trabecular pattern of mineralization in the growth plate involves rod-like mineralized struts of cartilage that run the longitudinal length of the skeletal element alongside columns of hypertrophic chondrocytes (Sawae et al., 2003; Jaroszewicz et al., 2016). During endochondral ossification, mineralized cartilage also can serve as a scaffold for the formation of trabecular-patterned endochondral bone (Gerstenfeld and Shapiro, 1996; Rauch, 2005; Touaitahuata et al., 2014), but the patterning relationships between these events are unclear.

Chondrocytes are the scleroblast type that form vertebrate cartilage, and the process of cartilage formation (chondrogenesis) is exemplified during endochondral ossification. During chondrogenesis, mesenchymal cells are converted to chondrocytes with a very transient chondroblast stage, because as soon as they begin to differentiate, they immerse themselves immediately in cartilage ECM, which contains abundant type 2 collagen (Col2) (Goldring et al., 2006). During endochondral ossification, chondrocytes form the cartilage template for subsequent bone formation and undergo a specific process called chondrocyte maturation (Eames et al., 2003). Morphologically, chondrocyte maturation includes hypertrophy (i.e., increase in cell size) and mineralization of the cartilage ECM, such as noted above in growth plates. Perhaps in an identical fashion to osteoblasts, mature chondrocytes secrete vesicles that initiate cartilage ECM mineralization (Anderson, 2003; Bottini et al., 2018). Molecularly, expression of Runt-related transcription factor 2 (Runx2) and Indian hedgehog (Ihh) in mature chondrocytes links developing cartilage to surrounding bone (Lefebvre and Smits, 2005). Runx2 induces *Ihh* expression in mature chondrocytes, and Ihh diffuses to adjacent perichondral cells, inducing differentiation of osteoblasts to form compact-patterned perichondral bone (Lefebvre and Smits, 2005; Komori, 2011). We will discuss the possibility that these basic skeletal biology concepts, largely derived from studies of tetrapods, apply to chondrichthyan endoskeletal tissues below.

## What Histological Regions of Tesserae Produce Tesseral Mineralization Patterns?

Tesserae are a defining feature of the extant chondrichthyan endoskeleton, and recent work leads toward a new view on how mineralization patterns of tesserae are organized in discrete histological compartments. Traditionally, tesserae were described as a distinctive polygonal mineralization pattern in chondrichthyans that is unique among vertebrates (Figures 1D,E; Kemp and Westrin, 1979; Maisey, 2013; Maisey et al., 2021). This polygonal mineralization pattern forms a superficial tiled structure beneath the perichondrium (i.e., subperichondral) of chondrichthyan endoskeletal elements. In addition to the polygonal pattern, recent work on endoskeletal tissues in Eaton’s and little skates revealed a previously-unappreciated trabecular mineralization pattern (Atake et al., 2019).

All chondrichthyan tesserae do not necessarily exhibit a polygonal mineralization pattern, but they all appear to exhibit a trabecular mineralization pattern, characterized by branching, rod-like struts (Figure 1F; Atake et al., 2019). The trabecular mineralization pattern (also described as stellate) can occur either underlying the polygonal mineralization pattern or can occur independently in the absence of the traditional polygonal mineralization pattern (Atake et al., 2019; Jayasankar et al., 2020). Large unmineralized regions between mineralized rod-like struts clearly distinguish the trabecular mineralization pattern from the polygonal mineralization pattern (Figures 1E,F; Atake et al., 2019). Of note, the exact mineralization patterns of tesserae can vary among endoskeletal elements in the same animal; sometimes the patterns even vary within different regions of the same skeletal element. One example of many is the little skate vertebra, the transverse processes of which exhibit polygonal and trabecular tesseral mineralization patterns, while the neural spine of the vertebra exhibits the trabecular pattern only (Figures 1D–F). Recent work highlighting the importance of mechanical forces in shaping the morphology and function of chondrichthyan tesserae might shed light on what actually generates this dimorphism of mineralization patterns (Jayasankar et al., 2020; Seidel et al., 2021).

**FIGURE 2:**
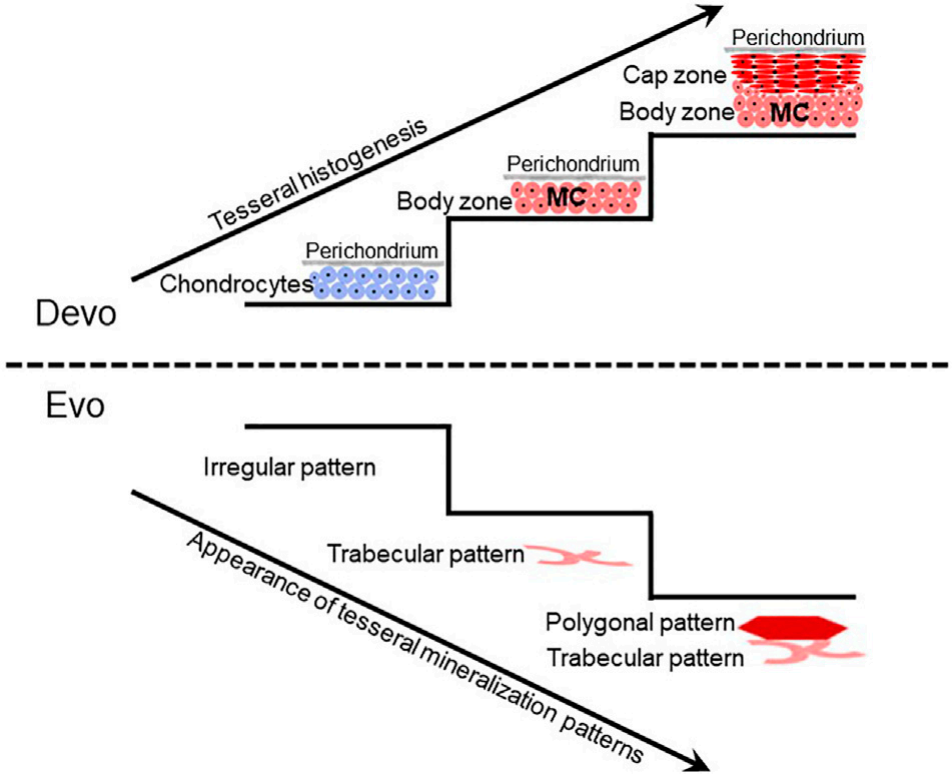
Tesseral development might reflect the evolution of tesseral mineralization patterns. Current limited data suggest that during tesseral development, the body zone of mineralized cartilage (MC) develops first, and the cap zone develops later atop the body zone. The fossil record can test whether this developmental sequence reflects the evolutionary history of tesseral mineralization patterns. Irregular patterns of mineralized cartilage in some acanthodians suggest that ill-defined mineralization patterns preceded well-defined trabecular and polygonal tesseral mineralization patterns in modern chondrichthyans. The emergence of the polygonal tesseral pattern after the trabecular tesseral pattern both in development and evolution needs to be ascertained.

Histological and molecular analyses of tesserae help clarify the nature of the tissues underlying tesseral mineralization patterns. There are two histological regions in tesserae: the cap zone and the body zone (Kemp and Westrin, 1979). The cap zone is subperichondral while the body zone underlies the cap zone (Figure 2). As we discuss in more detail below, cells within the cap zone of tesserae have morphological similarities to osteocytes and seem to secrete Col1 (Kemp and Westrin, 1979; Seidel et al., 2016; Seidel et al., 2017; Atake et al., 2019). By contrast, the body zone of tesserae consists of chondrocytes with round lacunae, large, mineral-dense, acellular regions termed spokes, and a Col2-rich ECM (Enault et al., 2015; Seidel et al., 2017). Prismatic mineralization and globular mineralization are traditional paleontological terms that distinguish biomineralization in the cap zone and body zone, respectively. Prismatic mineralization in the cap zone involves lime-prisms, while globular mineralization in the body zone involves globules of mineralized cartilage enriched in Liesegang lines and acellular spokes (Ørvig, 1951; Kemp and Westrin, 1979; Ørvig, 1989; Dean and Summers, 2006; Seidel et al., 2016). These histological features highlight two distinct regions in tesserae: a cap zone that exhibits bony features and a body zone that contains unmineralized and mineralized cartilage.

What regions of the tesserae actually produce these two tesseral mineralization patterns? The polygonal mineralization pattern in sharks and skates appears to occur subperichondrally, exclusively within the cap zone (Seidel et al., 2016; Maisey et al., 2021). Current data are not conclusive regarding how the trabecular mineralization pattern relates to histological features of tesserae. The cap zone of polygonal tesserae is much larger than the cap zone of trabecular tesserae (Atake et al., 2019). When occurring in association with the polygonal pattern, the trabecular pattern either derives from the deep portion of the cap zone or mineralized cartilage in the body zone. When occurring in isolation, the trabecular pattern might derive from the small cap zone or mineralized cartilage in the body zone (Atake et al., 2019). Again, the radiating pattern of acellular, mineral-dense spokes of the deep zone suggest that spokes are strong candidates for the trabecular mineralization pattern (Figure 1F; Seidel et al., 2016). Careful measurements correlating mineralized portions with histology can resolve between these two possibilities.

## Developmental Studies of Chondrichthyan Tesserae can Shed Light on Mineralization Pattern Evolution

Recent studies have begun to clarify how morphological features of tesserae are conserved or vary across chondrichthyan clades. Most extant chondrichthyan studies have focussed on elasmobranchs, where polygonal tesserae are widespread (Kemp and Westrin, 1979; Maisey, 2013; Seidel et al., 2016; Seidel et al., 2017; Atake et al., 2019; Marrama et al., 2019). Trabecular (or stellate) tesserae have not been widely described using those terms, but various studies suggest that this mineralization pattern is also widespread among extant elasmobranchs and some fossil chondrichthyans. For example, trabecular/stellate tesserae are present in the propterygium of the round stingray *Urobatis halleri* and in the cranium of the stem-holocephalan *Cladoselache* (Frey et al., 2019; Jayasankar et al., 2020; Maisey et al., 2021).

Studies of the skeleton in chimaeras were extremely limited until the past couple of years, but these recent analyses are providing much-needed data to understand the evolution of the “classical” chondrichthyan trait of tesserae. For example, tesserae in the synarcual of the adult elephant shark *Callorhinchus milii* and in the chondrocranium of the adult rabbit fish *Chimaera monstrosa* do not exhibit either trabecular or polygonal mineralization patterns, instead showing an irregular mineralization pattern (Pears et al., 2020; Seidel et al., 2020). It is unclear which region of tesserae creates the irregular mineralization pattern, because “classical” features of tesserae, such as the cap and body zones, are not discernable. However, histological analyses show that tesserae in chimaeras appear acellular, which is a characteristic of the body zone in elasmobranch tesserae (Debiais-Thibaud, 2018; Pears et al., 2020; Seidel et al., 2020).

Despite these limited data on extant chimaeras, fossil data suggest that the last common ancestor of extant chondrichthyans had polygonal and trabecular tesserae. For example, tesserae in the braincase of fossil chimaeras exhibit polygonal and trabecular mineralization patterns, but their histological features are not known (Finarelli and Coates, 2014; Coates et al., 2017; Pears et al., 2020). Furthermore, recent analyses of tesseral features in fossil chondrichthyans suggest that ill-defined (irregular) patterns of mineralized cartilage, such as those in some acanthodians, might be evolutionary precursors of well-defined trabecular and polygonal mineralization patterns in modern chondrichthyans (Figure 2; Burrow et al., 2018; Maisey et al., 2021).

**FIGURE 3:**
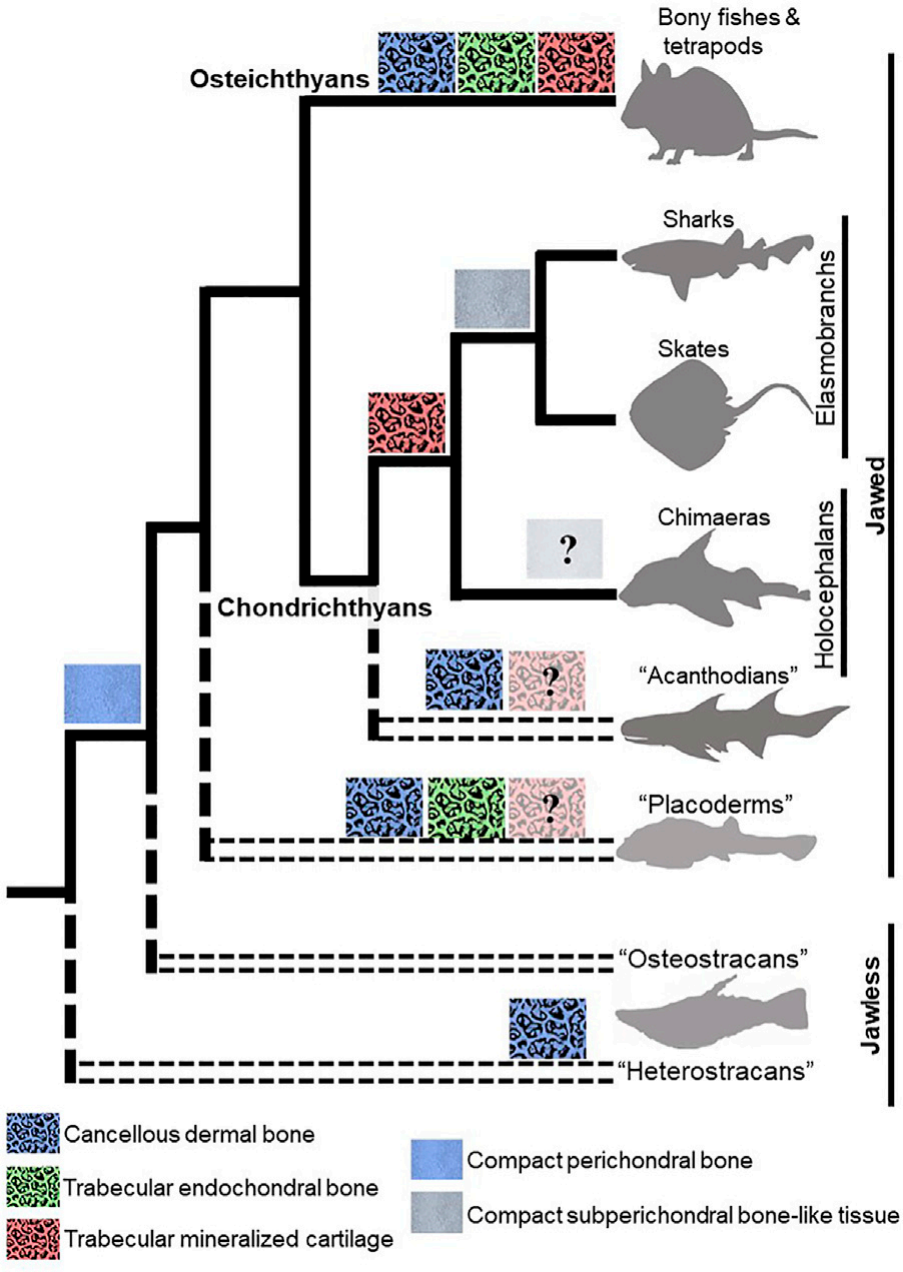
Evolutionary appearances of mineralization patterns of bone and cartilage in vertebrates. Dermal bones in fossil and extant jawless and jawed vertebrates have a cancellous microstructure that is similar to that of trabecular endochondral bone. The recently-described trabecular mineralization pattern of cartilage in chondrichthyans argues that a trabecular mineralization pattern is an ancestral feature of vertebrates, so the presence of trabecular mineralized cartilage should be re-evaluated in ancestral jawless and jawed vertebrates. Compact subperichondral bone-like tissue is an elasmobranch synapomorphy, but endoskeletal tissues in chimaeras need to be examined to ascertain whether this feature would be symplesiomorphic in extant chondrichthyans. The presence of compact perichondral bone in the ancestors of chondrichthyans and osteichthyans suggest that subperichondral bone-like tissue and perichondral bone are homologous. Paraphyletic groups are represented by doubled, dashed lines.

Fossil data argue that the trabecular mineralization pattern recently described in extant chondrichthyans actually appeared very far back during vertebrate evolution. Radiating “trabecles of mineralized cartilage” have been described in tesserae of the stem-elasmobranch *Palaeobates polaris* (Figure 3; Ørvig, 1951). Dermal skeletal tissues, such as dentine and dermal bones (i.e., those forming from intramembranous ossification), in the exoskeleton of jawless fishes (e.g., heterostracans) and jawed fishes (e.g., placoderms, acanthodians, and osteichthyans) have a cancellous microstructure similar to the trabecular mineralization pattern (Figure 3; Ørvig, 1951; Smith and Hall, 1990; Sanchez et al., 2012; Giles et al., 2013; Keating et al., 2015). The trabecular mineralization pattern in the vertebrate endoskeleton is commonly illustrated by endochondral bone (i.e., bone deposited within a degrading cartilage template), which was long argued to appear first in osteichthyans (Donoghue and Sansom, 2002; Donoghue et al., 2006). New fossil data describe endochondral bone with a trabecular mineralization pattern also in placoderm-like fish (Brazeau et al., 2020), suggesting that the trabecular mineralization pattern is present in the endoskeleton of ancestral vertebrates (Figure 3). Given that ostracoderms and placoderms also had mineralized cartilage in their endoskeletons, further work should clarify whether a trabecular mineralization pattern in cartilage was pervasive in these ancestral vertebrates.

Perhaps reflecting shared ancestry, the trabecular mineralization pattern of chondrichthyan tesserae and trabecular bone of other vertebrates share morphological features. In fact, we named the trabecular mineralization pattern of chondrichthyan tesserae after the well-described trabecular bone (i.e., true endochondral bone) that forms during endochondral ossification. Both trabecular tesserae and endochondral bone share morphological similarities, such as trabecular struts and unmineralized regions between them (Figures 1C,F). The average thickness of trabecular struts is a relatively constant measure of endochondral bone in tetrapods (Mullender et al., 1996; Swartz et al., 1998; Holzer et al., 2012; Tsegai et al., 2017). Interestingly, trabecular thickness is indistinguishable between trabecular mineralization patterns of chondrichthyan tesserae and endochondral bone in tetrapods (Atake et al., 2019). While both examples of trabecular mineralization patterns have unmineralized regions between them, those regions contain fat (and marrow in tetrapods) in endochondral bone and cartilage in trabecular tesserae. In addition, the trabecular pattern of endochondral bone projects in three dimensions, while the trabecular pattern of trabecular tesserae only extends in two dimensions. Despite these differences, similarities in the mineralization patterns of trabecular tesserae and trabecular bone suggest that, if these mineralization patterns are not homologous, then at least the same set of genes dictating the trabecular patterning process might have been co-opted during evolution of these tissues.

Comparing the development of the trabecular mineralization patterns of endochondral bone and trabecular tesserae will no doubt help to assess any homology between them. Mineralized cartilage is the scaffold upon which endochondral bone is formed, but how endochondral bone derives its trabecular mineralization pattern remains unclear. During endochondral ossification, cartilage ECM in the hypertrophic zone of the growth plate is organized into longitudinal and transverse septa; ECM of the longitudinal septa becomes mineralized, while the transverse septa are unmineralized (Gerstenfeld and Shapiro, 1996; Sawae et al., 2003; Jaroszewicz et al., 2016). Sometimes, trabeculae of mineralized cartilage even persist after cartilage ECM degradation, and osteoblasts deposit bone matrix on these cartilage remnants to form endochondral bone (Rauch, 2005; Touaitahuata et al., 2014). Despite common misunderstanding in the skeletal biology field, studies demonstrate clearly that matrix degradation of growth plate cartilage occurs by proteolytic activity of vascular endothelial cells, not chondro-/osteoclasts (Lewinson and Silbermann, 1992; Lee et al., 1995; Romeo et al., 2019). Molecularly, the Notch pathway in endothelial cells appears to drive the pattern of cartilage degradation, and thus Notch signalling is the only known molecular determinant of trabecular bone patterning (Ramasamy et al., 2014). While previous studies of this process were in 2D (Lewinson and Silbermann, 1992; Gerstenfeld and Shapiro, 1996; Sawae et al., 2003), recent studies using high resolution 3D images will provide unique insights into the trabecular patterning mechanism of endochondral bone (Ramasamy et al., 2014; Jaroszewicz et al., 2016).

Developmental studies of tesserae might reveal evolution of chondrichthyan mineralization patterns. Unfortunately, only limited studies on chondrichthyan skeletal development have been published (Lorch, 1949; Jollie, 1971; Reif, 1980; Miyake et al., 1992; Dahn et al., 2007; Eames et al., 2007; Dean et al., 2009; Gillis et al., 2009a; Gillis et al., 2009b; Gillis et al., 2011; Johanson et al., 2013; Johanson et al., 2015; O’Shaughnessy et al., 2015; Seidel et al., 2016; Criswell et al., 2017; Johanson et al., 2019; Smith et al., 2019; Marconi et al., 2020; Pears et al., 2020; Smith et al., 2020), and none of these has looked carefully at tesseral development. During development of polygonal tesserae in the round stingray, an early stage is deposition of islets of globular mineralized cartilage (Seidel et al., 2016). Given that the body zone of adult tesserae contains globular mineralization, these data support previous speculation that the body zone precedes the cap zone during tesseral development (Figure 2; Kemp and Westrin, 1979; Ørvig, 1989). If the trabecular mineralization pattern derives from the body zone, then it would be interesting to reveal if the trabecular mineralization pattern precedes the polygonal mineralization pattern during development. Such a finding might shed light on the evolution of these two mineralization patterns, since traits that appear earlier in development often appear earlier in evolution (Figure 2; De Beer, 1930; Haeckel, 1866; Ørvig, 1989). In addition, homology of trabecular mineralization patterns of tesserae and endochondral bone would be strengthened by analyses of Notch signalling during tesseral development.

## The Elasmobranch Centrum Exhibits a Unique Areolar Mineralization Pattern

In the centrum (mineralized portion of the vertebral body) of sharks and skates, mineralization occurs in concentric rings of the perichordal sheath surrounding the notochord in what is termed an areolar mineralization pattern (Ridewood and MacBride, 1921; Ørvig, 1951; Dean and Summers, 2006). While the areolar mineralization pattern is considered one of the hallmarks of the chondrichthyan endoskeleton, it has never been described in the centra of chimaeras. Unconstricted mineralized perichordal sheaths have been described in some chimaeras (Gadow and Abbott, 1895; Didier, 1995), but whether they reflect the areolar mineralization pattern and its histological features is yet to be clearly ascertained.

An elastic interna, a middle fibrous sheath, and an elastic externa form the perichordal sheath, which supports the development and mineralization of the centrum (for a review of the different types of vertebral centra, see Gadow and Abbott, 1895; Arratia et al., 2001). During development, migrating mesenchymal cells that will form cartilage of the vertebral body are thought to constrict the perichordal sheath, giving the elasmobranch centrum a biconcave morphology (Figure 1G; Gadow and Abbott, 1895). Some mesenchymal cells actually invade the perichordal sheath and help to differentiate the middle fibrous sheath into three distinct layers: inner, middle, and outer (Ridewood and MacBride, 1921; Eames et al., 2007; Criswell et al., 2017). Rounded cell lacunae of the inner and outer centrum layers reflect chondrocytes embedded in cartilage matrix. Cells of the middle centrum layer, which have elongated cell lacunae, are thought to produce the areolar mineralization pattern.

Centra in both elasmobranchs and ray-finned fishes demonstrate a biconcave morphology (Gadow and Abbott, 1895; Laerm, 1976), but they might not be homologous. Like elasmobranch centra, centra in ray-finned fishes also derive their biconcave morphology from the constriction of the perichordal sheath by migrating mesenchymal cells (Gadow and Abbott, 1895). Unlike centrum development in elasmobranchs, however, mesenchymal cells do not invade the perichordal sheath during centrum development in ray-finned fishes (Gadow and Abbott, 1895; Grotmol et al., 2003). Consequently, biconcave centra in elasmobranchs and ray-finned fishes derive from cellular and acellular mineralization of the perichordal sheath, respectively. This difference in the developmental processes of biconcave centra in elasmobranchs and ray-finned fishes has made their homology contentious, and despite tremendous similarities in morphology, centra in these two animal groups are thought to have evolved independently (Arratia et al., 2001; Grotmol et al., 2003; Fleming et al., 2015; Maisey et al., 2021). Developmental differences also suggest that centra evolved independently in different lineages of ray-finned fishes (Laerm, 1982; López-Arbarello and Sferco, 2018).

## Chondrichthyan Subperichondral Bone-Like Tissues Might Be Homologous to Perichondral Bone

Bone is one of the novelties characterizing vertebrate evolution (Hyman, 1943; Zhang and Cohn, 2008), and bone was widespread among vertebrates prior to the evolution of jaws (Stensiö, 1927; Janvier, 1990; Smith and Hall, 1990; Min and Janvier, 1998; Keating et al., 2015). Furthermore, the two main extant jawed vertebrate groups, chondrichthyans and osteichthyans, diverged from a common bony ancestor about 420 million years ago (Inoue et al., 2010). Acanthodians have long been recognized as a group of extinct jawed vertebrates with endoskeletal bone, and analyses over the past decade have led to the understanding that some members of this group are actually stem chondrichthyans (Davis et al., 2012; Brazeau and de Winter 2015; Maisey et al., 2021). So, both ancestral jawed vertebrates and stem chondrichthyans had endoskeletal bone. What about living chondrichthyans, which are thought to retain the most ancestral vertebrate features (Criswell and Gillis, 2020; Hirschberger et al., 2021)? Despite all of these phylogenetic data, the chondrichthyan endoskeleton has traditionally been characterized as lacking bone (Ørvig, 1951; Zangerl, 1966; Moss, 1977; Janvier, 1981; Maisey, 1988; Clement, 1992). Like chondrichthyans, Acipenseriformes (sturgeons and paddlefishes) have a predominantly cartilaginous endoskeleton and retain features of ancestral vertebrates (Cheng et al., 2020). The presence of endoskeletal bone in Acipenseriformes is also yet to be clearly demonstrated despite reports of mineralized bone-like tissue in Siberian sturgeons (Leprévost et al., 2017; Warth et al., 2017).

Current data lead to the idea that extant chondrichthyans might not have lost the endoskeletal bone that was present in their ancestors. Two locations within the elasmobranch endoskeleton have received much attention in this respect: the neural arches, which are dorsal extensions from the vertebral body that protect the neural tube (Arratia et al., 2001), and the cap zone of tesserae. Specifically, subperichondral neural arch tissue and the cap zone of tesserae (hereafter referred to as subperichondral bone-like tissues) show histological and molecular features that are consistent with bone. Back in 1932, Wurmbach observed that subperichondral neural arch tissue of some sharks is compact and develops appositionally (Wurmbach, 1932). Subsequent work supported this finding, not only in many species of shark, but also in skates and other batoids, suggesting that this bone-like tissue in the neural arch might at least be an elasmobranch synapomorphy (Figure 1H; Figure 3; Eames et al., 2007; Atake et al., 2019; Berio et al., 2021). Data on chimaeras are needed in order to understand whether this might be an ancestral trait of all living chondrichthyans. Similarly, Kemp and Westrin proposed that the cap zone of tesserae might also be bone-like (Kemp and Westrin, 1979). The mineralization pattern in polygonal tesserae of both sharks and skates is compact, and as discussed above, new data on chimaera tesserae suggest that they have a somewhat compact mineralization pattern (Pears et al., 2020; Seidel et al., 2020). In sum, morphological data demonstrate that both tesserae and neural arches exhibit bone-like features, and such features might have been present in the last common ancestor to extant chondrichthyans.

Cell morphological and limited molecular studies also support the idea that extant chondrichthyans make endoskeletal bone. In typical vertebrate perichondral bone, osteocyte lacunae demonstrate an elongate morphology, and bone ECM has high levels of Col1 (Rossert and de Crombrugghe, 2002; Currey, 2003; Atkins and Findlay, 2012). Indeed, cell lacunae in chondrichthyan subperichondral bone-like tissues also exhibit an elongate morphology, and histological, immunohistochemical, and electron microscopy analyses show the presence of tightly packed Col1 in the ECM of subperichondral bone-like tissues (Kemp and Westrin, 1979; Peignoux-Deville et al., 1982; Eames et al., 2007; Seidel et al., 2017). Interestingly, similar to osteocyte canaliculi, cell lacunae in subperichondral neural arch tissue of the catshark might be connected by nano-channels (Bordat, 1987). Bone is a metabolically active tissue that undergoes remodelling (Hadjidakis and Androulakis, 2006). While *Sox9/Sox5/Sox6*-expressing perichondral cells were shown recently to mediate cartilage regeneration in the little skate (Marconi et al., 2020), the capability of chondrichthyan bone-like tissues to undergo (cellular- or acellular-mediated) remodelling is currently untested. Nevertheless, morphological, histological, and molecular features suggest that chondrichthyan subperichondral bone-like tissues and perichondral bone are homologous.

Two criteria must be met for chondrichthyan subperichondral bone-like tissues to be homologous with perichondral bone: shared ancestry and shared developmental programs. Homologous characters are classically defined by descent from a common ancestor (Jardine, 1967). Clearly, the last common ancestor of chondrichthyans and osteichthyans had perichondral bone (Figure 3; Ørvig, 1951; Donoghue and Sansom, 2002; Donoghue et al., 2006; Maisey, 2013), so extant chondrichthyan subperichondral bone-like tissues might be a modified perichondral bone. Homology of chondrichthyan subperichondral bone-like tissues and perichondral bone should be further assessed by comparative embryology, because homologous characters must share a developmental program, even though each clade might have modified that ancestral program independently (Boyden, 1947; Sachs, 1982; Van Valen, 1982; Roth, 1984; Stevens, 1984; Tomlinson, 1984; Wagner, 1989; Roth, 1991; Gilbert and Bolker, 2001). Perichondral bone formation has been well-studied, but only limited features of chondrichthyan neural arch or tesseral development have been described (Eames et al., 2007; Seidel et al., 2016), so research is desperately needed on the development of chondrichthyan bone-like tissues.

**FIGURE 4:**
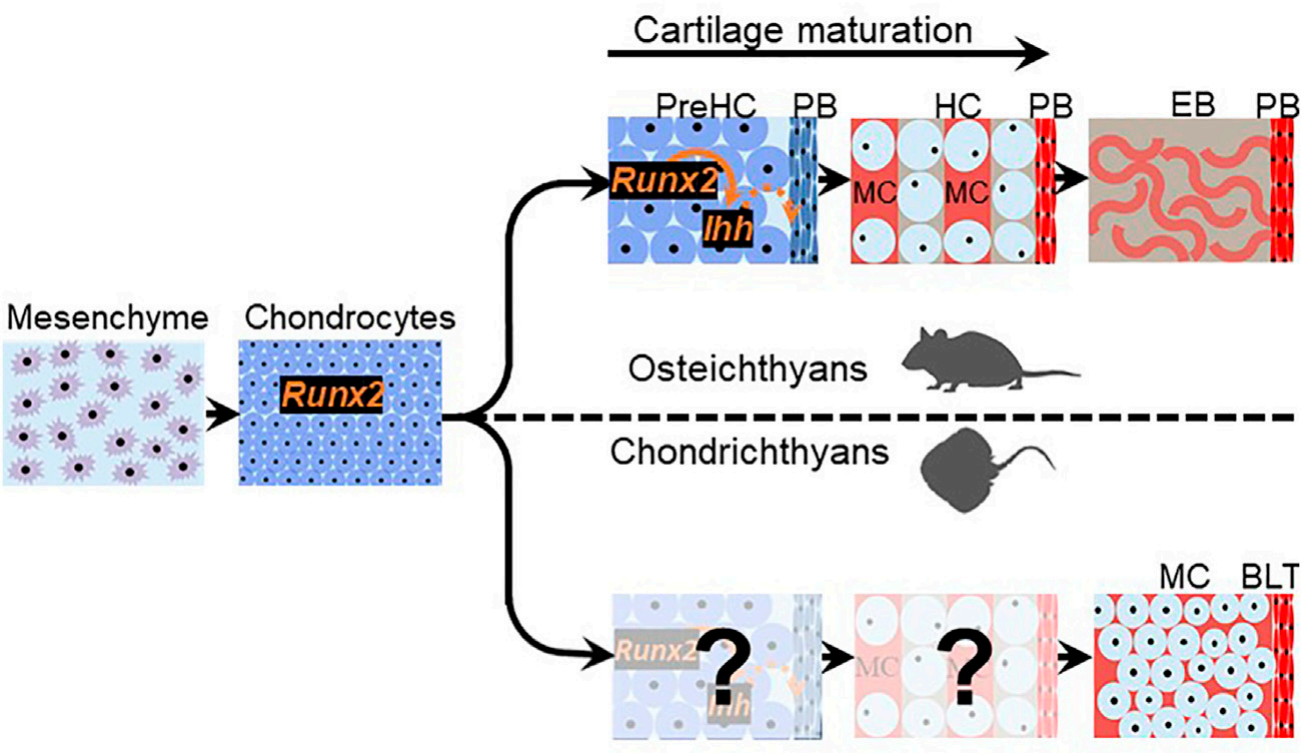
Chondrichthyan subperichondral bone-like tissues and osteichthyan perichondral bone might share a developmental program. Chondrichthyan subperichondral bone-like tissue (BLT) and osteichthyan perichondral bone (PB) develop in association with a cartilage template that expresses *Runx2*. In osteichthyans, the cartilage template undergoes a maturation process involving the creation of mature hypertrophic chondrocytes (HC) and expression of *Ihh*, which induces perichondral bone. If cartilage maturation occurs during the development of chondrichthyan subperichondral bone-like tissues, then the persistence of mineralized cartilage (MC) in chondrichthyans (and early vertebrates outside the gnathostome crown group such as placoderms and ostracoderms) and the additional cartilage degradation step occurring in osteichthyans argues that cartilage maturation is evolvable.

If chondrichthyan subperichondral bone-like tissues and perichondral bone were homologous, then what developmental features might they share? Chondrichthyan subperichondral bone-like tissues appear to develop in association with a cartilage template (Eames et al., 2007), so that aspect seems conserved with perichondral bone (Figure 4). Runx2 and Ihh expression during cartilage maturation is required for induction of adjacent perichondral bone in osteichthyans (Figure 4; Lanske et al., 1996; Vortkamp et al., 1996; Hoshi et al., 1999; Inada et al., 1999; Kim et al., 1999; St-Jacques et al., 1999; Long et al., 2001; Kronenberg, 2003; Hammond and Schulte-Merker, 2009; Eames et al., 2011). Interestingly, *Runx2* is expressed in developing cartilages of the dogfish shark, and the ability of Runx proteins to induce Hedgehog genes might be an ancestral trait of all chordates (Hecht et al., 2008). However, it remains unclear what molecules drive the differentiation of chondrichthyan subperichondral bone-like tissues. Furthermore, perichondral bone in osteichthyans derives from perichondral osteoprogenitor cells, while some true endochondral bone derives from mature chondrocytes *trans*-differentiating into osteoblasts (Moskalewski and Malejczyk, 1989; Roach, 1992; Hammond and Schulte-Merker, 2009; Zhou et al., 2014; Giovannone et al., 2019). Whether chondrichthyan bone-like tissues derive from the perichondrium or from chondrocyte *trans*-differentiation is unknown.

To summarize, in order to assess correspondence of the developmental programs of chondrichthyan subperichondral bone-like tissues and perichondral bone, some key aspects of chondrichthyan endoskeletal development must be revealed:

1) Do chondrocytes in the cartilage template of chondrichthyan subperichondral bone-like tissues undergo hypertrophy and express maturation genes, such as *Runx2* and *Ihh*?
2) Does Hedgehog signalling induce bone-like tissues?
3) Do chondrichthyan subperichondral bone-like tissues derive from the perichondrium and/or chondrocytes?

More broadly, elucidating the process of perichondral ossification in ancestral vertebrates would be key to understanding the evolutionary history of perichondral ossification. However, direct assessment of the developmental process of perichondral bone in extinct ancestral jawless and jawed vertebrates is not feasible, unless many fossilized embryos and larvae of different stages are identified, such as the remarkable discoveries of ptyctodontid placoderm embryos and acanthodian larvae (Zidek, 1985; Long et al., 2008; Chevrinais et al., 2017). Also, living jawless vertebrates (lampreys and hagfishes) do not mineralize their skeleton (Shimeld and Donoghue, 2012). Thus, the basal phylogenetic position of chondrichthyans makes their subperichondral bone-like tissues an excellent proxy for assessing the mechanism of perichondral ossification in ancestral vertebrates. In jawless (e.g., osteostracans) and jawed (e.g., placoderms) vertebrates, perichondral bone typically overlies a persistent mineralized cartilage (Ørvig, 1951; Wang et al., 2005; Long et al., 2015). Similarly, subperichondral bone-like tissues in chondrichthyans overlie a persistent mineralized cartilage, which would be the body zone in the case of tesserae and the cartilage core in the case of the neural arches (Figure 2; Eames et al., 2007; Seidel et al., 2016; Atake et al., 2019). These data argue that the process of cartilage maturation is highly evolvable as mineralized cartilage persists (and induces perichondral bone?) in stem-pan-gnathostomes (e.g., heterostracans and placoderms), whereas a subsequent cartilage degradation step occurs in crown-gnathostomes (e.g., osteichthyans) to facilitate endochondral bone formation (Figure 4).

## Using Comparative Transcriptomics to Test for Chondrichthyan Bone

If chondrichthyan bone-like tissues and perichondral bone are homologous, then they would derive from a homologous cell type: the osteoblast. Homologous cell types are evolutionary units defined by descent from a common ancestor (Arendt, 2008; Arendt et al., 2016). Each cell type expresses a characteristic set of genes, termed a molecular fingerprint, which can be conserved across phylogenetic lineages (Sudmant et al., 2015; Liang et al., 2018).

A few candidate genes have been used conventionally to characterize evolution of skeletal cell types, but this approach is very limited. For example, Collagen type 10 alpha 1 (*Col10a1*) is expressed by mature chondrocytes and osteoblasts in teleosts and other ray-finned fishes, including medaka, zebrafish, and spotted gar (Laue et al., 2008; Albertson et al., 2010; Renn and Winkler, 2010; Eames et al., 2012). Only one of six identified duplicates of *Col10a1* in the catshark is expressed specifically by cells in subperichondral neural arch tissue (Debiais-Thibaud et al., 2019). This shared expression of a given gene by osteoblasts, mature chondrocytes, and cells of bone-like tissue highlights some of the limitations in adopting candidate gene approaches to demonstrate cell type homology. Are cells that form chondrichthyan bone-like tissue best characterized as osteoblasts or mineralizing chondrocytes?

Comparative transcriptomics of osteoblasts, mineralizing chondrocytes, and cells forming chondrichthyan bone-like tissues can resolve the molecular fingerprints of cells forming chondrichthyan subperichondral bone-like tissues. Unbiassed transcriptomic profiling of cell types is readily achievable following the advent of deep RNA sequencing. The molecular fingerprint should be revealed by RNA sequencing of a specific cell type at a developmental stage when differentiation genes are highly expressed (Arendt, 2008). Cells that form chondrichthyan bone-like tissues would be best characterized as osteoblasts if they demonstrate the osteoblast molecular fingerprint. However, osteoblasts can evolve in clade-specific fashions (Nguyen and Eames, 2020), so what, if anything, is the vertebrate osteoblast molecular fingerprint to which chondrichthyan data should be compared?

Defining the osteoblast molecular fingerprint must involve transcriptomic profiling of osteoblasts from several vertebrate groups. In addition to unravelling a conserved suite of genes, these data need to be coupled to phylogenetic bioinformatic analyses to estimate the ancestral osteoblast molecular fingerprint. A survey of unbiased transcriptomic profiles of osteoblasts reveals that transcripts have been mainly from studies on mammals (Ayturk, 2019). More efforts are therefore needed to uncover the transcriptome of osteoblasts in non-mammalian vertebrates to enable a comprehensive definition of the vertebrate osteoblast molecular fingerprint. In addition, bioinformatics techniques need to be developed to compare networks of transcriptomic data quantitatively and infer ancestral gene networks (for a recent review, see Ovens et al., 2021).

## Conclusion

The designation of chondrichthyans as “cartilaginous fishes” was clearly made at a time when research tools were very limited. The advent of high-resolution imaging and contemporary molecular techniques has renewed investigative interests on morphological and molecular features of the chondrichthyan endoskeleton. Unraveling the developmental mechanisms underlying the formation of tesserae and subperichondral bone-like tissues generally will provide tremendous insight into the evolutionary history of endochondral and perichondral bone among vertebrates, guiding future investigations on vertebrate mineralized skeletal tissues. Employing comparative embryology and transcriptomics can robustly test the hypothesis that the chondrichthyan endoskeleton lacks bone. The question “Do sharks and relatives make bone?” has lasted for many centuries, but for the first time the answers are now within experimental reach.
